# Aerobic biogenesis of selenium nanoparticles by *Enterobacter cloacae* Z0206 as a consequence of fumarate reductase mediated selenite reduction

**DOI:** 10.1038/s41598-017-03558-3

**Published:** 2017-06-12

**Authors:** Deguang Song, Xiaoxiao Li, Yuanzhi Cheng, Xiao Xiao, Zeqing Lu, Yizhen Wang, Fengqin Wang

**Affiliations:** Institute of Feed Science, Zhejiang University, 866 Yuhang Tang Road, Hangzhou, 310058 China

## Abstract

In the present study, we examined the ability of *Enterobacter cloacae* Z0206 to reduce toxic sodium selenite and mechanism of this process. *E. cloacae* Z0206 was found to completely reduce up to 10 mM selenite to elemental selenium (Se°) and form selenium nanoparticles (SeNPs) under aerobic conditions. The selenite reducing effector of *E. cloacae* Z0206 cell was to be a membrane-localized enzyme. iTRAQ proteomic analysis revealed that selenite induced a significant increase in the expression of fumarate reductase. Furthermore, the addition of fumarate to the broth and knockout of *fumarate reductase* (*frd*) both significantly decreased the selenite reduction rate, which revealed a previously unrecognized role of *E. cloacae* Z0206 fumarate reductase in selenite reduction. In contrast, glutathione-mediated Painter-type reactions were not the main pathway of selenite reducing. In conclusion, *E. cloacae* Z0206 effectively reduced selenite to Se° using fumarate reductase and formed SeNPs; this capability may be employed to develop a bioreactor for treating Se pollution and for the biosynthesis of SeNPs in the future.

## Introduction

Selenium (Se) is an important element for life and exhibits redox activity in the environment^1^. The Se cycle (see Figure S1, in Supplementary Information) is complex because the element can exist in a variety of oxidation states, ranging from −II to + VI^2, 3^. Se is released into the environment either from the weathering of Se-rich rocks^2, 4^ (e.g., black shales, carbonaceous, limestones, carbonaceous cherts, mudstones, and seleniferous coal) or from anthropogenic sources from industrial and agricultural activities^5^. Se can exist in the environment in multiple forms, including ionic selenate or selenite, solid-state Se(0), and selenocysteine/selenoproteins^6^. The toxicity rank of these forms is selenite > selenocysteine > selenate ≈ selenomethionine > elemental Se^7–10^. Apart from natural Se originating from weathering of seleniferous soils and rocks, anthropogenic activities, e.g. mining, metal refining and coal fire-based power production, lead to Se contamination in the environment^11^. Thus, remediation measures are required to treat Se contamination, because it has become an important public health concern^12^. At present, physicochemical methods, e.g. nanofiltration, reverse osmosis, ion exchange, ferrihydrite and zero valent iron, are usually used for Se removal from waste water. However, such physicochemical methods are commonly high-cost or inefficient for selenium removal^13^.

The lifetime of selenite in soils is closely associated with microbial activity^11^. Certain strains that are resistant to selenite and reduce selenite to the Se° or to methylated Se forms^14–18^, may potentially be used for the bioremediation of contaminated soils, sediments, industrial effluents, and agricultural drainage waters. The ABMet^®^ Technology developed by GE Water & Process Technologies efficiently removes selenate and selenite from waste water via bacteria reduction, and the elemental Se could be separated from the biofilter tank through a backwash process^11^. It is worth noting that most bacterially assembled Se° particles are selenium nanoparticles (SeNPs), which are deposited inside a cell (cytoplasmic), within the periplasm or extracellularly^12, 19–21^. These particulate SeNPs display special physical characteristics, such as photoelectric, semiconducting and X-ray sensing properties^11, 22^. They also possess an adsorptive ability, antioxidant functions, and due to their high surface area-to-volume ratio, marked biological reactivity^23–26^. However, there is now growing concern about the environmental impact of nanoparticle synthesis based on physico-chemical methods that require high pressures and temperatures, are energy consuming, use toxic chemicals, and generate hazardous byproducts^27^. Consequently, applications using biological systems such as microbial cultures for the production of metal nanoparticles, including SeNPs, are becoming an increasingly realistic alternative^27, 28^.

Reduction of selenite to Se° has been shown to be mediated by thiols (Painter-type reactions) in the cytoplasm as part of a microbial detoxification strategy^29^. Selenite reacts with GSH and forms selenodiglutathione (GS-Se-SG), which is further reduced to glutathione selenopersulfide (GS-Se^−^) by NADPH-glutathione reductase. GS-Se^−^ is an unstable intermediate and undergoes a hydrolysis reaction to form Se° and reduced GSH. In addition to Painter-type reactions, a number of terminal reductases for anaerobic respiration, two nitrite reductases, an inducible sulfite reductase and a fumarate reductase, have also been reported to be able to carry out selenite reduction in cells^30–33^.

*Enterobacter cloacae* SLD1a-1, a selenate-respiring facultative anaerobe, has been demonstrated to catalyze the reduction of both selenate and selenite to Se°^12, 34, 35^, but the selenite or selenate concentrations adopted in these studies were extremely low (less than 1.5 mM). The reduction of selenate was shown to be mediated by a membrane-bound molybdoenzyme^36, 37^, but the mechanism of selenite reduction in this strain has not been elucidated. Moreover, all of the selenite-reducing assays involving *E. cloacae* in these studies were performed in an anaerobic environment, and the selenite-reducing ability of *E. cloacae* has not previously been investigated under aerobic conditions. *E. cloacae* Z0206, a strain that we isolated from Reishi mushroom (*Ganoderma lucidum*) “meat”^38^, was found to possess excellent selenite resistance, tolerating more than 100 mM selenite. In the present investigation, we studied (i) the selenite-reducing ability of *E. cloacae* Z0206 under aerobic conditions, (ii) the characteristics and location of the produced SeNPs, and (iii) the mechanism of selenite reduction in the Z0206 strain.

## Results and Discussion

### Growth profile and selenite-reducing ability of *E. cloacae* Z0206 under different selenite concentrations

**Figure 1:**
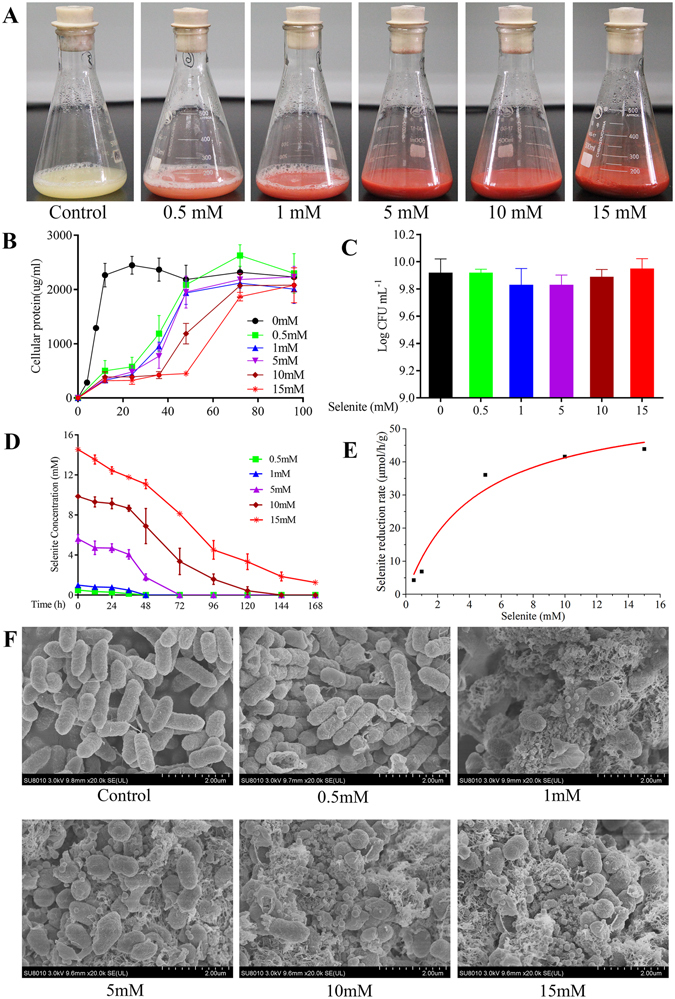
Growth and selenite reduction of *E. cloacae* Z0206 in the presence of various concentrations of selenite. (**A**) Apparent changes in spent broth. (**B**) Growth profile under different selenite concentrations. Samples of 1 ml of the bacterial culture were collected at different time intervals of bacteria growth and then centrifuged at 4 °C and 10,000 × *g* for 10 min. Protein was extracted from the pellet using a total bacterial protein extraction assay kit. Bacterial growth was measured via the quantification of total cell protein. The protein concentration in bacteria cell extracts was determined. (**C**) Quantity of bacteria at the time point of 96 h. (**D**) Dynamic changes in selenite residue in the broth. (**E**) Kinetic study of selenite reduction by *E. cloacae* Z0206 under different selenite concentrations. Line graphs and bar graphs are presented as the mean ± SD, **P < 0.01 (n = 3). (**F**) SEM analysis of *E. cloacae* Z0206 under different selenite concentrations. The bacteria were cultured in the presence of various concentrations of selenite (0 mM, 0.5 mM, 1 mM, 5 mM, 10 mM and 15 mM). Samples were collected when the selenite was completely consumed according to the results in Fig. 1D.

To determine the toxicity of selenite to the microorganism, the growth profile of *E. cloacae* Z0206 was studied under various concentrations of selenite (0, 0.5, 1, 5, 10, 15 mM). According to the apparent changes in the spent broth shown in Fig. 1A, we found that the strain formed a reddish cell suspension, which indicated its ability to reduce the toxic, colorless, soluble selenite ions to the non-toxic, red, insoluble elemental form of Se (Se°). It is worth noting that the red color of the broth darkened, and viscosity increased with the increase of the selenite concentration. The results regarding the growth profile (Fig. 1B) showed that the addition of selenite strikingly inhibited the growth rate of *E. cloacae* Z0206, and the inhibitory effect strengthened with the increase of the selenite concentration during the log phase. However, the final cell density in the presence of selenite (0.5 mM–15 mM) was comparable to that of the control without selenite addition, as verified by the result of a bacteria counting assay performed at 96 h (Fig. 1C). Evaluation of the selenite-reducing ability of the bacterium (Fig. 1D) showed that selenite was rapidly reduced by this strain, with 10 mM selenite being completely reduced in 144 h. As shown in Fig. 1E, the rates of selenite reduction were modeled using the Michaelis-Menten kinetic equation (see section 1.1, in Supplementary Information). A nonlinear least-square analysis of the data yield a *K*_m_ value of 4.37 mM and a *V*_max_ of 59.32 μmol/h/g. Scanning electron microscopy (SEM) analysis of the morphology of the bacterium and reduced selenite (Fig. 1F) revealed that as the selenite concentration increased, the rod-shaped cells tended to become shorter. Selenite at concentrations greater than or equal to 1 mM significantly stimulated the secretion of extracellular polymeric substances. SeNPs ranging from 100–300 nm were observed scattered around the cells and occurred as aggregates attached to the bacterial biomass in the presence of selenite, and the particle density grew with the increase of the selenite concentration. These results indicated that selenite merely reduced the growth rate of *E. cloacae* Z0206 rather than decreasing the final amount of bacteria. Additionally, the bacterium detoxified selenite by rapidly reducing it to Se° and formed SeNPs, highlighting the species as a promising exploitable option for setting up of low-cost biological treatment units for the bioremediation of Se-laden effluents.

### Characterization and localization of SeNPs in Z0206 cultures

**Figure 2:**
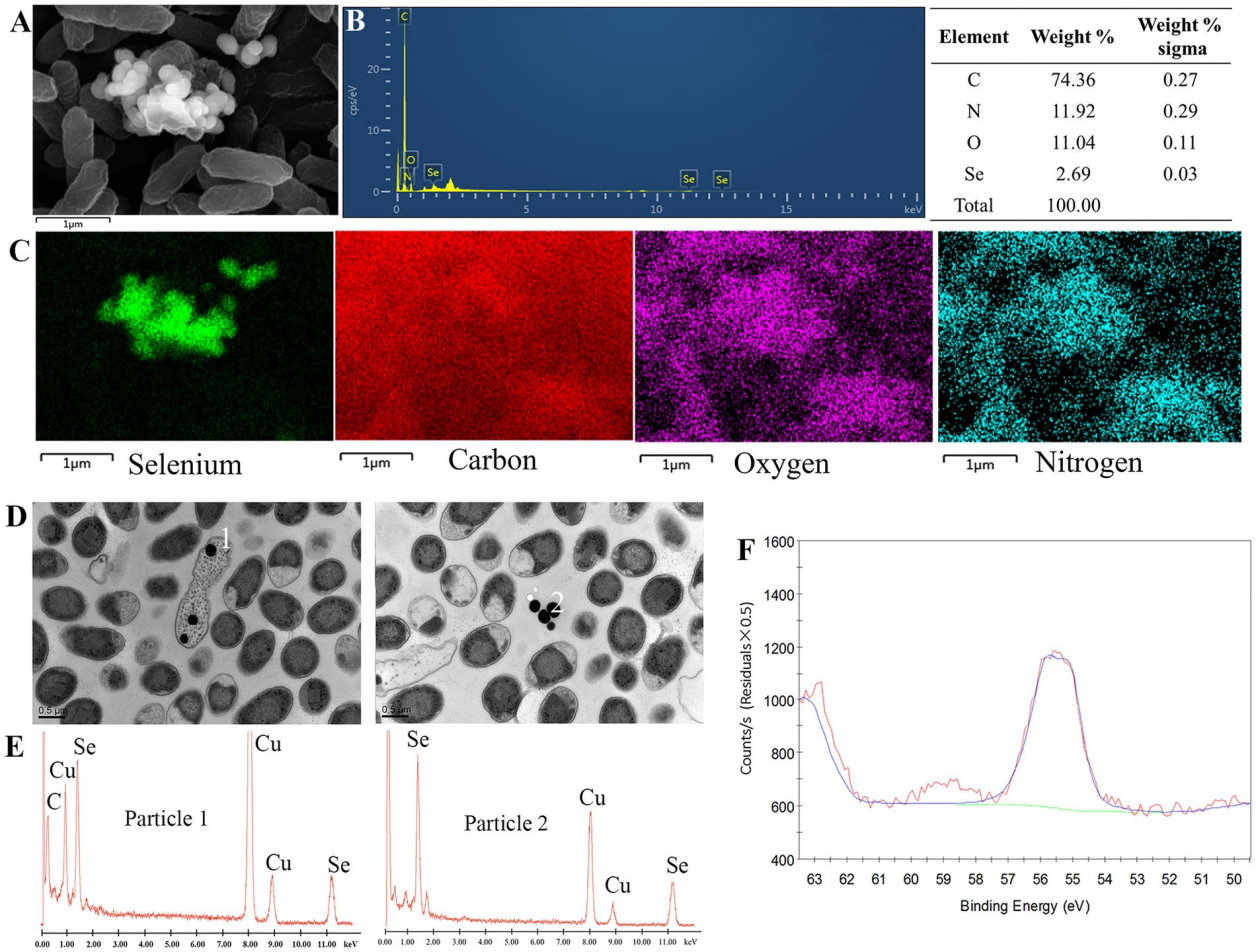
Characterization and localization of *E. cloacae* Z0206 synthesized SeNPs. (**A**) SEM image of *E. cloacae* Z0206 and SeNPs. (**B**) EDX analysis of the contents of carbon, nitrogen, oxygen and selenium in the area of (**A**). (**C**) Elemental mapping analysis of the distribution of selenium, carbon, oxygen and nitrogen in the area of (**A**). (**D**) TEM images of *E. cloacae* Z0206 and SeNPs. (**E**) EDX analysis of particle 1 and 2 in (**D**). (**F**) High-resolution Se 3D XPS of purified SeNPs synthesized by *E. cloacae* Z0206.

Energy Dispersive X-ray Spectrum (EDX) flat scanning of the area in Fig. 2A revealed a strong Se atom signal, accounting for 2.69% of the total component elements (Fig. 2B). EDX elemental mapping was used to detect the Se distribution. Four elemental maps of Se, carbon, oxygen and nitrogen were obtained and are shown in different colors based on the scanning area encompassing both the contents of *E. cloacae* Z0206 and the surrounding area (Fig. 2C). The map of elemental oxygen and nitrogen showed the cell shape and distribution of biomass. In contrast, element carbon was distributed both within and outside of cells because the cells were embedded by carbon-containing Epon plastic. However, the Se strong signals, shown in green, perfectly matched the profile of the extracellular nanoparticles, verifying that these particles were SeNPs. Moreover, Se, oxygen and nitrogen overlapped in the SeNPs distribution area, implying that these SeNPs may be coated with biomass. In addition, there was a weak Se signal inside the bacteria, suggesting that SeNPs may also exist inside the cells. However, Transmission electron microscopy (TEM) analysis revealed the presence of electron-dense nanoparticles both within and outside of the cells (Fig. 2D), which was not found in cell cultures without selenite (see Figure S2, in Supplementary Information). The EDX spectra of these nanospheres clearly indicated the presence of Se, as specific absorption peaks at 1.37 and 11.22 keV were recorded (Fig. 2E). The lack of peaks corresponding to other metals indicated that Se occurred in its elemental state (Se°) rather than as a metal selenide. This was confirmed by X-ray photoelectron spectroscopy (XPS) analysis, which shows clearly the 3D spectral peak of Se° (Fig. 2F). These results suggested that *E. cloacae* Z0206 reduces Se(IV) to Se(0) and assembles it into nanoparticles.

### Selenite-reducing ability of different cellular fractions of *E. cloacae* Z0206 cells

**Figure 3:**
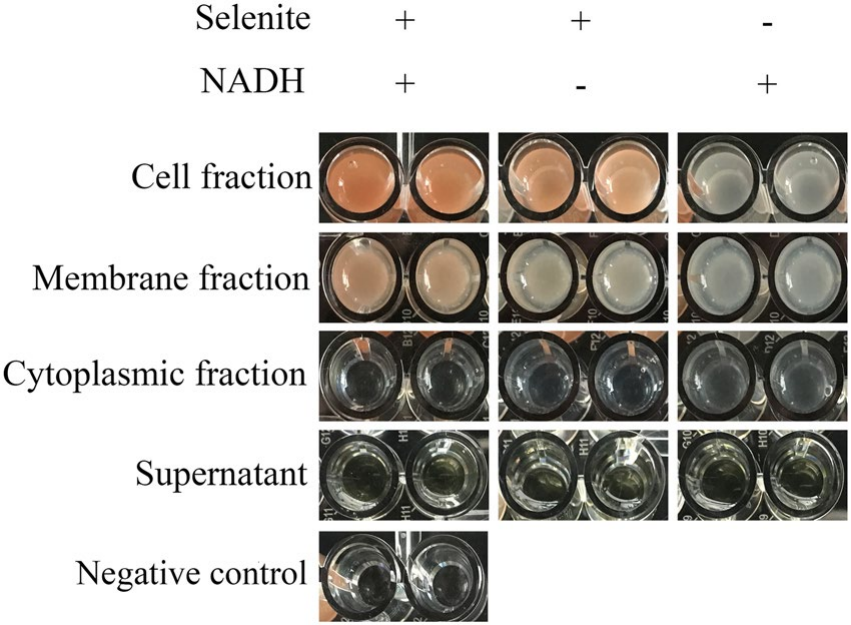
Selenite-reducing ability of different fractions of *E. cloacae* Z0206. The selenite-reducing ability of the bacterium was estimated using the following reaction mixture: 100 µg protein, 10 mM selenite and 10 mM NADH in 200 µl Tris-HCl (pH 7.5). The reaction mixture was incubated at 37 °C for 8 h. The reaction mixture with the total cell fraction and without selenite served as positive and negative controls, respectively.

Different mechanisms have been proposed for the reduction of selenite to Se° in microorganisms, including (i) Painter-type reactions^29^, (ii) the thioredoxin reductase system^39^, (iii) siderophore-mediated reduction^40^, (iv) sulfide-mediated reduction^6^ and (v) dissimilatory reduction^30–32^. Among these potential mechanisms, i, ii and v occur inside the cell, while the other reactions occur extracellularly. To help determine how selenite was reduced by *E. cloacae* Z0206, subcellular fractions, including cytoplasm, membrane proteins and the supernatant from liquid cultures, were isolated after 12 h of growth without selenite, and the selenite-reducing ability of each fraction was evaluated. As shown in Fig. 3, selenite was reduced to orange-colored Se by only the membrane-associated proteins after the addition of NADH, whereas little orange-colored Se occurred without NADH. However, boiling the membrane fraction samples resulted in a complete loss of reduction activity (data not shown). These results indicated that the reduction process mediated by membrane-associated proteins was an enzymatic reaction and was NADH dependent. Interestingly, the cytoplasmic fraction and supernatant failed to change the color to orange, suggesting that these two fractions possess no selenite-reducing ability.

### iTRAQ analysis of *E. cloacae* Z0206 proteins in response to selenite

It is well established that *α*, *β* and *γ* proteobacteria possess high GSH levels in the cytoplasm, which drive Painter-type reduction to reduce selenite^29^. Although *E. cloacae* Z0206 is a *γ* proteobacterium, the above results indicated that it may reduce selenite through a membrane-bound enzyme rather than through a thiol-related reaction. To identify the probable mechanism involved, we undertook a large-scale proteomic analysis (iTRAQ) to examine the modification of protein expression in response to selenite (complete data on protein quantification are shown in Table S1, in Supplementary Information). The analysis focused on the proteins whose expression varied by more than a factor of 1.2. According to this criterion, 172 proteins were induced after selenite treatment, and 212 proteins were significantly repressed. All of the significantly differentially regulated proteins identified were subjected to gene ontology analysis. Among the 287 proteins involved in biological processes, 274 proteins and 175 proteins were dedicated to metabolic processes and cellular processes, respectively. For each of the three ontologies, the annotated data revealed that the proteins were mainly distributed among two or three of the general term categories. In the biological process category, the three most abundant categories included proteins that are involved in metabolic processes (244), cellular processes (230), and single-organism processes (198). In the molecular process category, the two main categories involved in catalytic activity (207) and binding (172). In the cellular component category, the three largest categories were cell (153), macromolecular complex (63), and membrane (61) (see Figure S3, in Supplementary Information).

Among the identified proteins, selenite induced a 2.42-fold increase in fumarate reductase abundance (Table 1). Li, *et al*.^33^ demonstrated that the reduction of selenite in *Shewanella oneidensis* MR-1 is mediated by fumarate reductase, indicating that fumarate reductase may play a role in the selenite-reducing process in *E. cloacae* Z0206. However, the most expected antioxidant proteins, such as glutathione synthetase, glutathione reductase, glutathione-disulfide reductase, thioredoxin, thioredoxin reductase and thioredoxin-dependent thiol peroxidase, showed no significant change in response to selenite treatment (see Table S2, Support Information).

**Table 1:** Identification of proteins induced by selenite (>2.0-fold).

Description	Fold	Significance
Tagatose-bisphosphate aldolase	3.78	7.20E-50
Cytochrome D ubiquinol oxidase subunit I	2.92	7.16E-33
DNA polymerase V subunit UmuC	2.86	9.93E-32
Damage-inducible protein I	2.81	1.16E-30
DNA repair protein RecN	2.80	1.53E-30
Virulence protein MsgA (Fragment)	2.68	4.44E-28
Damage-inducible protein YebG	2.54	2.93E-25
Integrase	2.53	3.23E-25
60 kDa heat shock protein (Fragment)	2.51	7.83E-25
Fumarate reductase subunit C	2.42	6.52E-23
Na + dependent nucleoside transporter domain protein	2.35	1.41E-21
Small heat shock protein IbpA	2.19	1.86E-18
Fructose-bisphosphate aldolase	2.09	2.02E-16

### Analysis of the mRNA abundance of selected genes

**Figure 4:**
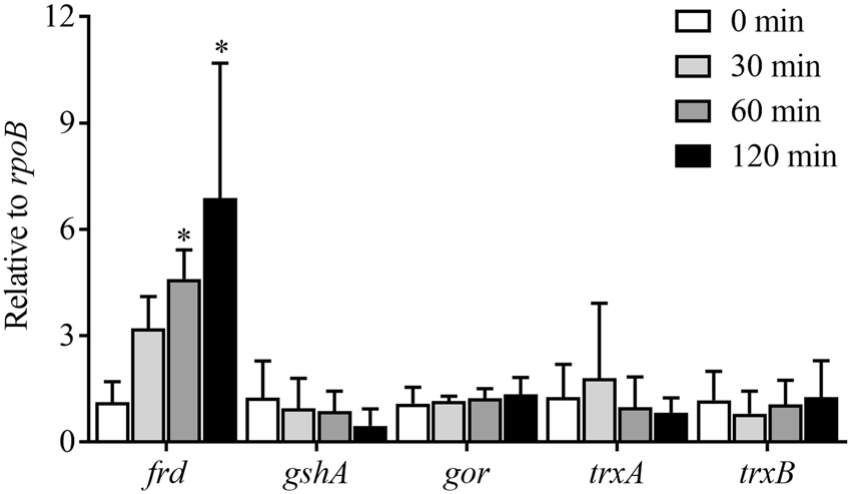
Effect of selenite on the transcription of fumarate reductase and enzymes involved in Painter-type reactions. An overnight culture of *E. cloacae* Z0206 was adjusted to OD_600_ = 1 and diluted into fresh broth (1%). When the culture reached the stationary phase, a 2 ml sample of the culture was removed (as a Se-free control). A 500 μL aliquot of the sodium selenite stock solution (1 M) was then added, and the culture was incubated for another 120 min. Samples were collected at 30 min, 60 min and 120 min after the addition of selenite. *P < 0.05, **P < 0.01 (n = 3).

To verify the results of the iTRAQ analysis, the mRNA abundance of the enzyme *fumarate reductase* (*frd*) and the antioxidative enzymes *glutathione synthetase* (*gsh*A), *glutathione reductase* (*gor*), *thioredoxin* (*trx*A) and *thioredoxin reductase* (*trx*B) was assessed. As shown in Fig. 4, the mRNA expression of *gsh*A, *gor*, *trx*A and *trx*B in cells after stimulation by selenite was not different from that before selenite treatment. However, selenite treatment promoted the mRNA expression of *frd* in a time-dependent manner. These data verified that *E. cloacae* Z0206 may reduce selenite to Se^0^ through fumarate reductase instead of via a GSH-mediated Painter-type reaction.

### Effect of buthionine sulfoximine (BSO) on the selenite reduction rate in *E. cloacae* Z0206

**Figure 5:**
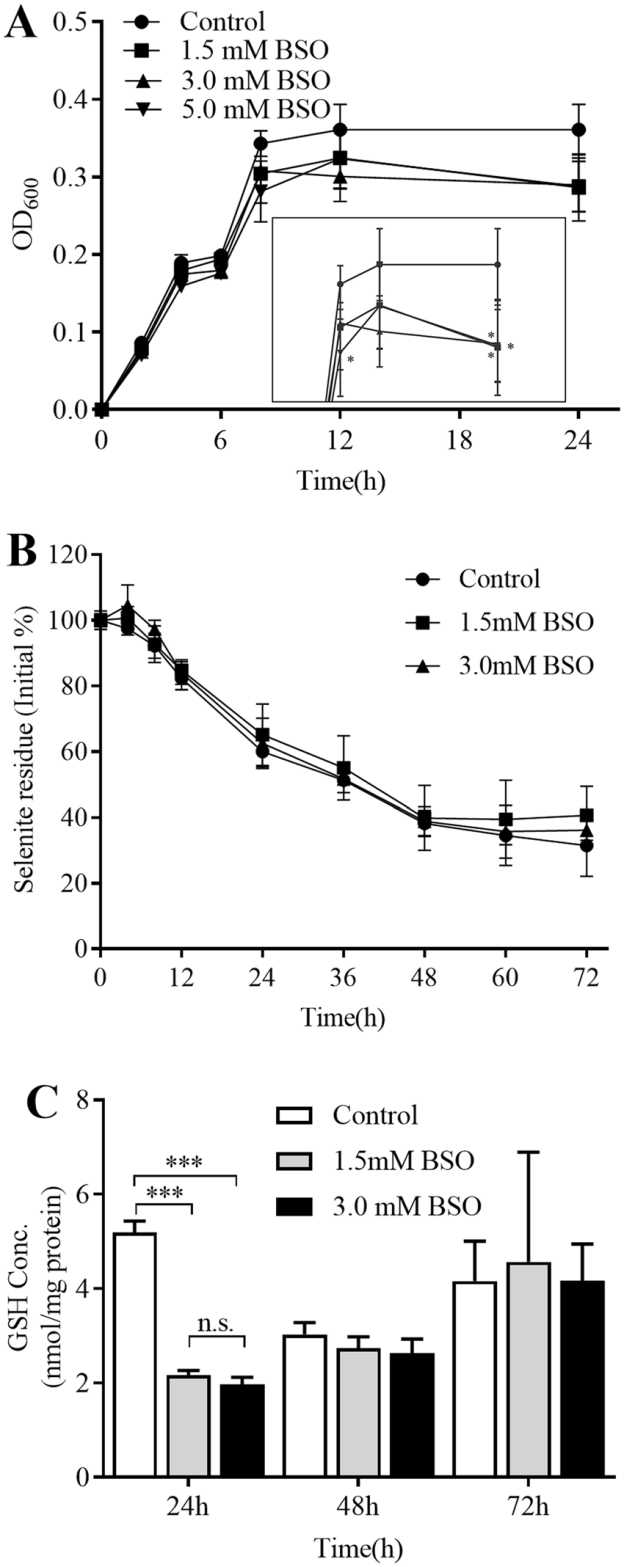
Effect of BSO on the growth and selenite reduction in *E. cloacae Z0206*. (**A**) Growth of *E. cloacae* Z0206 in the presence of 1.5 mM, 3.0 mM and 5.0 mM BSO. After that, Cells were incubated in the presence of 5 mM selenite while adding 0 mM, 1.5 mM or 3.0 mM BSO. Samples were collected at different time points to determine selenite residues, and cell samples were collected at 24 h, 48 h and 72 h to measure intracellular GSH concentrations. (**B**) Selenite reduction in the presence of BSO. The result was converted to a percentage of the initial selenite concentration. (**C**) Effect of BSO on intracellular GSH concentrations. *P < 0.05, ***P < 0.001 (n = 3).

To confirm that a GSH-mediated Painter-type reaction was not involved in the selenite-reducing process in *E. cloacae* Z0206, BSO, an inhibitor of glutathione synthetase, was used. As shown in Fig. 5A, BSO doses of 1.5 mM, 3.0 mM and 5.0 mM slightly decreased the growth rate during the exponential phase. In the stationary phase, the cell densities of all BSO-treated groups were slightly lower than that of the control group (all *P* < 0.05). Therefore, doses of 1.5 mM and 3.0 mM were chosen to study the effect of GSH on selenite reduction. As shown in Fig. 5B, the presence of 1.5 mM and 3.0 mM BSO did not result in any significant change in the selenite reduction rate. Z0206 cells were collected at 24 h, 48 h and 72 h to confirm the inhibitory effect of BSO on GSH synthesis (Fig. 5C). We found that the intracellular GSH concentration was decreased from 5.19 ± 0.24 to 2.16 ± 0.11 (1.5 mM BSO) and to 1.97 ± 0.15 (3.0 mM BSO) nmol/mg protein (both *P* < 0.001) at 24 h. This inhibition effect weakened and disappeared at 48 h and 72 h, respectively. However, the evidently lowered GSH concentration at 24 h did not lead to any significant change in the selenite reduction rate, indicating that a GSH-mediated Painter-type reaction was not the main pathway of selenite reduction in *E. cloacae* Z0206.

### Effect of fumarate on the selenite reduction rate in *E. cloacae* Z0206

**Figure 6:**
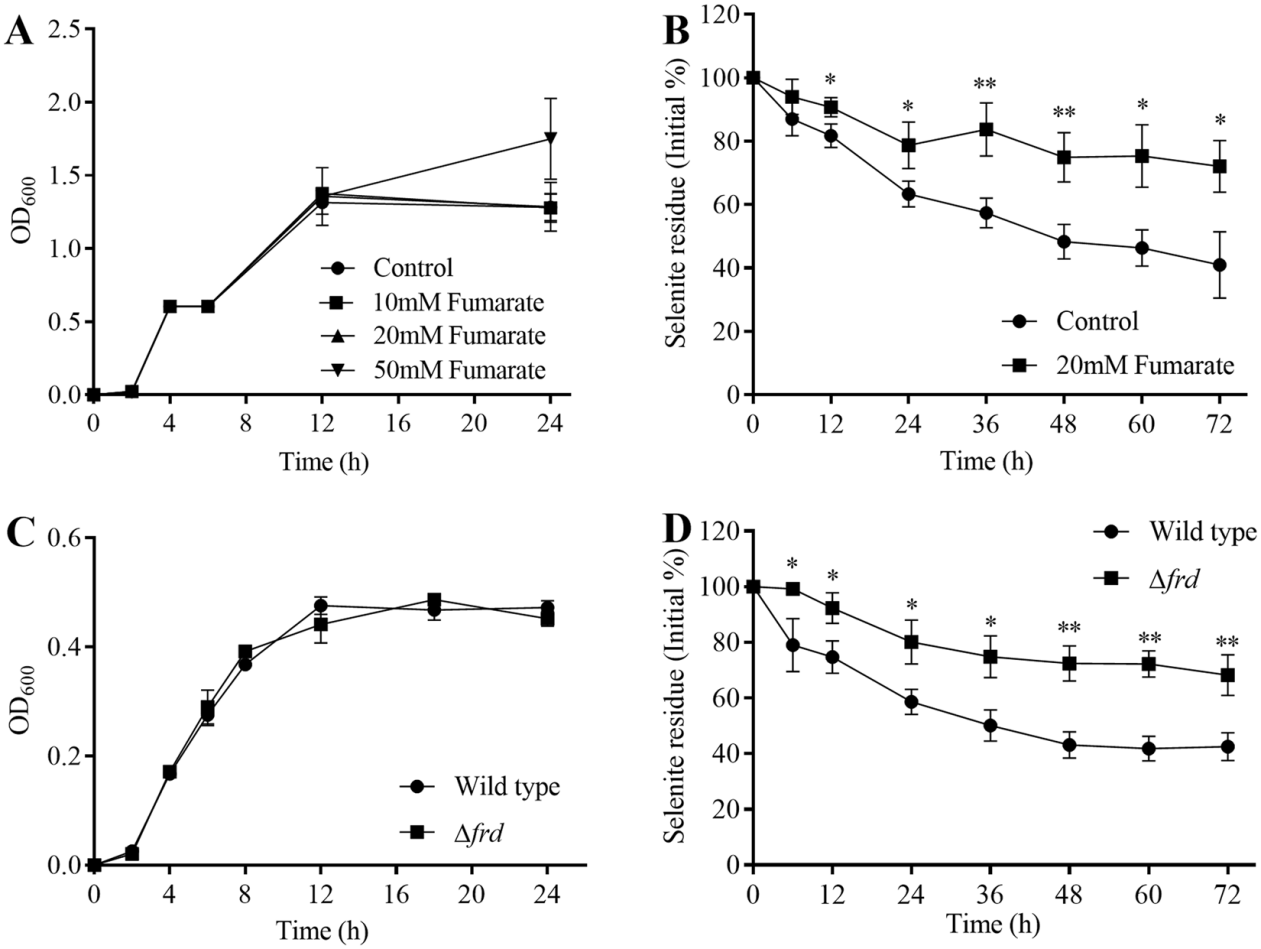
Effect of fumarate reductase on selenite reduction in *E. cloacae* Z0206. (**A**) Effect of fumarate on the growth of *E. cloacae* Z0206. The bacteria were cultured with the addition of 10 mM, 20 mM, or 50 mM fumarate or without the presence of fumarate; the OD_600_ values were measured at the indicated time point after 3-fold dilution. (**B**) Effect of fumarate on the selenite reduction rate. The bacteria were cultured in the presence of 5 mM selenite with or without 20 mM fumarate treatment. Samples were collected at different time points and centrifuged at 4 °C at 10,000 × *g* for 10 min, and the supernatant was used to determine the selenite residue. The selenite residue was converted to a percentage of the initial selenite concentration. (**C**) Effect of fumarate reductase mutation on the growth of *E. cloacae* Z0206. (**D**) Effect of fumarate reductase mutation on the selenite reduction rate in *E. cloacae* Z0206. *P < 0.05, **P < 0.01 (n = 3), compared with the selenite residue of the control group or the wild-type group at the same time.

The effect of fumarate on the selenite-reducing ability of the bacterium was evaluated to investigate whether selenite reduction in *E. cloacae* Z0206 was mediated by fumarate reductase. First, the impact of fumarate on the growth of this strain was studied. As shown in Fig. 6A, different concentrations of fumarate did not affect the growth rate during the exponential phase, whereas a dose of 50 mM led to an evident increase in cell density during the stationary phase. Thus, a dose of 20 mM fumarate was selected to ensure that the cell density was similar to that of control cells without fumarate treatment. Based on the results shown in Fig. 6B, a dose of 20 mM fumarate significantly decreased the reduction rate. After 72 h, 41% and 72% remaining selenite was detected in the cultures without and with fumarate, respectively. These results indicated that competition existed between selenite and fumarate for fumarate reductase^33^, and fumarate reductase may present the main pathway of selenite reduction in *E. cloacae* Z0206.

### Effect of fumarate reductase knockdown on the selenite-reducing ability of *E. cloacae* Z0206

We constructed the fumarate reductase-mutated strain Δ*frd* to confirm that enzyme’s role in selenite reduction in *E. cloacae* Z0206. As shown in Fig. 6C, mutation of fumarate reductase did not significantly influence the growth of the cell. However, the mutant strain Δ*frd* exhibited a markedly repressed selenite-reducing capacity compared with the wild-type strain. After 72 h of reduction, 68.25% of the original selenite remained in cultures of the Δ*frd* strain, while only 42.58% of selenite could be detected in cultures of the wild-type strain (Fig. 6D). Therefore, *E. cloacae* Z0206 reduces selenite mainly through fumarate reductase.

In conclusion, many bacterial strains have been demonstrated to reduce selenite to Se^0^ and form SeNPs under anaerobic conditions, whereas bacteria grown in aerobic conditions possess the ability to rapidly generate more bacterial cells within a short time period and under less stringent culture conditions. In the present study, *E. cloacae* Z0206 was found to effectively reduce selenite to Se^0^ under aerobic conditions, and form monodispersed nanosized (approximately 100–300 nm in diameter) particles, which were observed both within and outside of the cells. Moreover, the selenite-reducing factor of *E. cloacae* Z0206 was demonstrated to be a membrane-localized fumarate reductase rather than a GSH-mediated Painter-type reaction. Biosynthesis of SeNPs under aerobic conditions presents advantages over the chemical process, in which SeNPs are produced under environmentally harmful conditions. Thus, *E. cloacae* Z0206 may be used to develop a bioreactor for the treatment of Se pollution and biosynthesis of SeNPs. Further studies may focus on the properties of biogenic SeNPs, compared with chemically synthesized SeNPs and the potential applications in the fields of nanotechnology and biotechnology.

## Methods

### Bacterial strain Z0206 and culture conditions

*E. cloacae* Z0206, a strain that we previously isolated^38^, was cultured in an optimized broth (sucrose 25, tryptone 5, yeast extract 5, K_2_HPO_4_·3H_2_O 2.62, KH_2_PO_4_ 1, MgSO_4_ 0.5 in g L^−1^) at 32 °C and 250 rpm.

### Bacterial growth under selenite stress

The effect of selenite on the growth of *E. cloacae* Z0206 was determined in the presence of 0 mM, 0.5 mM, 1 mM, 5 mM, 10 mM and 15 mM sodium selenite. Sodium selenite was prepared as a 1 M stock solution and sterilized via filtration. Then, 500-ml Erlenmeyer flasks containing 100 ml broth was supplemented with increasing concentrations of selenite (0 mM, 0.5 mM, 1 mM, 5 mM, 10 mM and 15 mM), and an overnight-grown bacteria culture were adjusted to OD_600_ = 0.5 and inoculated (1% inoculum size) to the above mentioned broth containing various concentrations of selenite, followed incubation at 32 °C, 250 rpm for 96 h.

### Determination of selenite concentration

The culture was collected and centrifuged at 10,000 × *g* for 10 min. The supernatant was collected to detect residual selenite through the 2,3-diaminonaphthalene fluorimetric method^41^. Please see section 1.2 in Supplementary Information for introduction of the method to detect selenite.

### SEM and EDX analysis

Cultures of Z0206 in grown the presence of different concentrations of selenite (0 mM, 0.5 mM, 1 mM, 5 mM, 10 mM and 15 mM) were collected. These samples were centrifuged at 4 °C, 12,000 × *g* for 15 min, and then the pellets were washed (0.1 M PBS), fixed, dried and sputter-coated, followed by viewing under SEM. Elemental composition maps of selected areas were analyzed with the EDX system.

### TEM and EDX analysis

Cultures of Z0206 grown in the presence of various concentrations of selenite (0 mM, 0.5 mM, 1 mM, 5 mM, 10 mM and 15 mM) were collected. These samples were centrifuged at 4 °C, 12,000 × *g* for 15 min, and then the pellets were washed (0.1 M PBS), fixed, dried and embedded. Ultrathin sections of 100 nm were cut and stained, followed by viewing under TEM. The elemental composition of selected particles was analyzed using the EDX system.

### Se particle valence analysis

*E. cloacae* Z0206 was cultured in the presence of 5 mM selenite at 32 °C at 250 rpm for 72 h. Selenium particles were separated from the culture according to the protocol developed by Dobias, *et al*.^42^. with some modification. Briefly, the culture was decanted, followed by simple centrifugation at 4 °C at 8,000 ×*g* for 10 min to separate the suspended biomass. The collected Se particles present in the supernatant from the previous centrifugation step were concentrated via centrifugation at 4 °C at 20,000 ×*g* for 15 min. The pellet was then lyophilized and analyzed using XPS.

### Separation of cellular fractions and determination of selenite-reducing activity

An *E. cloacae* Z0206 culture grown to the exponential phase without selenite was collected and centrifuged at 4 °C at 10,000 × *g* for 10 min. The supernatant was then collected and filtered using a 0.2-µm filter. Next, the pellet was washed with 10 mM Tris-HCl (pH 7.5) twice and re-suspended in the same buffer for sonication, followed by centrifugation at 4 °C at 6,000 × *g* for 10 min to separate unbroken cells. Then, the supernatant was centrifuged at 4 °C at 25,000 × *g* for 40 min to separate the cytoplasm (supernatant) and membrane (pellet) fractions. The protein concentration was measured with a BCA assay kit.

### RT-PCR analysis

RNA was extracted using the RNeasy Protect Bacteria Mini Kit. Total RNA was subjected to a reverse transcription reaction using a Quanti Tect Reverse Transcription Kit. Quantitative PCR was performed with a StepOne Plus™ Real Time PCR System using a FastStart Universal SYBR Green Master (ROX).

### Determination of intracellular GSH concentrations

Cells, collected from the experiment on the “Effect of BSO on selenite reduction in *E. cloacae* Z0206” at the time points of 24 h, 48 h and 72 h, were concentrated via centrifugation at 10,000 × *g*, 4 °C for 10 min and resuspended in 50 mM Tris-HCl (pH 7.5), after which the cells were disrupted using sonication. The disrupted cells were centrifuged at 20,000 × *g* for 15 min, and the supernatants were collected to detect the concentration of GSH using a Total Glutathione Assay Kit.

### Construction of the Δ*frd* mutant

The Δ*frd* mutant was constructed as reported elsewhere^43^. Briefly, an *frd* gene fusion fragment was amplified and ligated via PCR, then ligated with pLP12 and subsequently transformed into *Escherichia coli* β2163. The resulting plasmids were introduced into *E. cloacae* Z0206 through conjugation with *E. coli* β2163. After two rounds of selection, the mutant carrying the *frd* gene deleted was validated through PCR using primers corresponding to sequences upstream and downstream of the deletion (see Figure S4, in Supplementary Information) and subsequent sequencing.

### Statistics

One-way analysis of variance (ANOVA) followed by an LSD multiple comparison test was used to determine the statistical significance for multiple comparisons, and Student’s *t*-test was used for pairwise comparisons. *P* < 0.05 was considered statistically significant. All statistical tests were carried out with SPSS 22 software. All data are expressed as the mean ± SD.

### Data availability

The data that support the findings of this study are available from the authors on reasonable request.

## Electronic supplementary material

Supplementary Materials

Dataset 1
