# Supplementary material for: Aerobic biogenesis of selenium nanoparticles by Enterobacter cloacae Z0206 as a consequence of fumarate reductase mediated selenite reduction
Source: Sci Rep. 2017 Jun 12;7:3239. doi: 10.1038/s41598-017-03558-3 (PMC5468319; doi:10.1038/s41598-017-03558-3)
Supplement: Supplementary file 1 — Supplementary Materials [file 41598_2017_3558_MOESM1_ESM.pdf]

Aerobic biogenesis of selenium nanoparticles by *Enterobacter cloacae* Z0206 as a consequence of fumarate reductase mediated selenite reduction

Deguang Song, Xiaoxiao Li, Yuanzhi Cheng, Xiao Xiao, Zeqing Lu, Yizhen Wang*, Fengqin Wang*

National Engineering Laboratory of Biological Feed Safety and Pollution Prevention and Control, Key Laboratory of Molecular Animal Nutrition, Ministry of Education, Key Laboratory of Animal Nutrition and Feed Science of Zhejiang Province, Institute of Feed Science, Zhejiang University, 866 Yuhang Tang Road, Hangzhou 310058, China

For correspondence: email wangfq@zju.edu.cn (Fengqin Wang) and yzwang321@zju.edu.cn (Yizhen Wang); Tel. +86 571 8898 2815; Fax +86 571 8898 2650.

1 *SI* Materials and Methods

1.1 Modeling approach of selenite reduction rate

The rates of selenite reduction were modeled using the Michaelis-Menten kinetic equation

$$\frac{d[SeO_3^{2-}]}{[m]\cdot dt}=\frac{V_{max}\cdot[SeO_3^{2-}]}{K_m+[SeO_3^{2-}]}$$

Where K_m is the half velocity constant (mM), V_{max} is the apparent maximum reduction rate (μmol/h/g), and [m] is the cellular protein concentration (g/L).

1.2 Determination of selenite concentration

1.2.1 Standard curve

Add 0.00, 0.50, 1.00, 2.00, 3.00, 4.00 and 5.00 ml Se standard working solution (0.2μg Se/ml) to 100 ml colorimetric tube with cover and add double distilled water to 30 ml. Adjust pH to 1.5-2 using hydrochloric acid. Add 3ml 25g/l hydroxylamine hydrochloride solution (containing 10g/l EDTA) and 3ml 1g/l DAN solution, followed by mixing. Heat these tubes at

100°C for 5 min and then cool them to ambient temperature. Add 10ml hexamethylene to each tube and vibrate for 1 min. After stratification, collect hexamethylene (upper layer) and measure fluorescence intensity at excitation and emission wavelengths of 376nm and 520nm using fluorescence spectrophotometer. Calculate the standard curve.

1.2.2 Determination of selenite residue

Add 1 ml sample to 100 ml colorimetric tube with cover and add double distilled water to 30 ml. The following step is same with section 1.2.1. Calculate the selenite concentration according to the standard curve.

1.3 Proteomic analysis (iTRAQ) of the effect of selenite on *E. cloacae* Z0206 protein expression

1.3.1 Sample preparation

An overnight culture of Z0206 was adjusted to OD_{600}=1 and inoculated into fresh broth (1%), followed by culture at 32°C at 250 rpm for 12 h. Stationary-phase growing cells were then cultured with or without 5 mM selenite for 2 h, after which 10 ml of each culture was collected and centrifuged at 4°C at 10,000 × *g* for 10 min. The pellet was washed twice with 10 mM Tris-HCl (pH 7.5). Finally, protein was separated from these pellets using a total bacterial protein extraction assay kit.

1.3.2. Filter-aided sample preparation (FASP digestion)

Protein (200 μg) from each sample was incorporated into 30 μl SDT buffer (4% SDS, 100 mM DTT, 150 mM Tris-HCl pH 8.0). The detergent, DTT and other low-molecular-weight components were removed using UA buffer (8 M urea, 150 mM Tris-HCl pH 8.0) by repeated ultrafiltration (Microcon units, 10 kD). Then, 100 μl iodoacetamide (100 mM IAA in UA buffer)

was added to block reduced cysteine residues, and the samples were incubated for 30 min in darkness. The filters were washed with 100 μl UA buffer three times and then with 100 μl dissolution buffer (DS buffer) twice. Finally, the protein suspensions were digested with 4 μg trypsin (Promega) in 40 μl DS buffer overnight at 37°C, and the resulting peptides were collected as a filtrate. The peptides from each sample were desalted on C18 Cartridges (Empore™ SPE Cartridges C18 (standard density), bed I.D. 7 mm, volume 3 ml, Sigma), concentrated via vacuum centrifugation and reconstituted in 40 μl of 0.1% (v/v) formic acid. The peptide content was estimated based on the UV light spectral density at 280 nm using an extinction coefficient of 1.1 for a 0.1% (g/l) solution, which was calculated on the basis of the frequency of tryptophan and tyrosine in vertebrate proteins.

1.3.3. iTRAQ labeling

The peptide mixture (100 μg) from each sample was labeled using the iTRAQ reagent [7] according to the manufacturer's instructions (Applied Biosystems).

1.3.4. Peptide fractionation via strong cation exchange (SCX) chromatography

iTRAQ-labeled peptides were fractionated through SCX chromatography using the AKTA Purifier system (GE Healthcare). The dried peptide mixture was reconstituted and acidified with buffer A (10 mM KH2PO4 in 25% of ACN, pH 3.0) and then loaded onto a PolySULFOETHYL 4.6 x 100 mm column (5 μm, 200 Å, PolyLC Inc, Maryland, U.S.A.). The peptides were eluted at a flow rate of 1 ml/min with a gradient of 0%–8% buffer B (500 mM KCl, 10 mM KH2PO4 in 25% of ACN, pH 3.0) for 22 min, 8–52% buffer B from 22-47 min, 52%–100% buffer B from 47-50 min, 100% buffer B from 50-58 min, and 0% buffer B after 58 min. Elution was monitored based on the absorbance at 214 nm, and fractions were collected

every 1 min. The collected fractions were desalted on C18 Cartridges (Empore™ SPE Cartridges C18 (standard density), bed I.D. 7 mm, volume 3 ml, Sigma) and concentrated by vacuum centrifugation.

1.3.5. Mass spectrometry

HPLC

Each fraction was injected for nano-LC-MS/MS analysis. The peptide mixture was loaded onto a reversed-phase trap column (Thermo Scientific Acclaim PepMap100, 100 μm*2 cm, nanoViper C18) connected to the C18 reversed-phase analytical column (Thermo Scientific Easy Column, 10 cm long, 75 μm inner diameter, 3 μm resin) in buffer A (0.1% formic acid) and separated with a linear gradient of buffer B (84% acetonitrile and 0.1% formic acid) at a flow rate of 300 nl/min controlled by IntelliFlow technology. The linear gradient was: 0-35% buffer B for 50 min, 35-100% buffer B for 5 min, then holding in 100% buffer B for 5 min.

LC-MS/MS analysis

LC-MS/MS analysis was performed on a Q Exactive mass spectrometer (Thermo Scientific) coupled to an Easy nLC system (Proxeon Biosystems, now Thermo Fisher Scientific) for 60 min. The mass spectrometer was operated in positive ion mode. MS data were acquired using the data-dependent top10 method, dynamically choosing the most abundant precursor ions from the survey scan (300–1800 *m*/*z*) for HCD fragmentation. The automatic gain control (AGC) target was set to 3e6, and the maximum inject time to 10 ms. The duration of dynamic exclusion was 40.0 s. Survey scans were acquired at a resolution of 70,000 at *m*/*z* 200, and the resolution for HCD spectra was set to 17,500 at *m*/*z* 200; the isolation width was 2 *m*/*z*. The normalized collision energy was 30 eV, and the underfill ratio, which specifies the minimum

percentage of the target value likely to be reached at the maximum fill time, was defined as 0.1%. The instrument was run with peptide recognition mode enabled.

1.3.6 Data analysis

MS/MS spectra were searched using the MASCOT engine (Matrix Science, London, UK; version 2.2) embedded in Proteome Discoverer 1.4. The parameters were set according to the table below.

Proteome Discover 1.4 parameters setting

Item	Value
Enzyme	Trypsin
Max Missed Cleavages	2
Fixed modifications	Carbamidomethyl (C), iTRAQ4/8plex (N-term), iTRAQ 4/8plex (K)
Variable modifications	Oxidation (M), iTRAQ 4/8plex (Y)
Peptide Mass Tolerance	± 20 ppm
Fragment Mass Tolerance	0.1Da
Database	See the project report
Database pattern	Decoy
Peptide FDR	≤0.01
Protein Quantification	The protein ratios are calculated as the median of only the unique peptides of the protein
Experimental Bias	Normalizes all peptide ratios based on the median protein ratio. The

	median protein ratio should be 1 after normalization.

1.4 Design of primers for RT-PCR analysis

The gene-specific primers were designed using the Prime Quest Tool at http://sg.idtdna.com/primerquest/Home/Index and shown in table below.

PCR prime sequences used in this study.

Gene	Description		Sequence (5’-3’)
frd	fumarate reductase	F	TCCAGACCTCCCTTCAGTT
		R	GACCGTCATCGACCAGAATATC
*gsh*A	glutathione synthetase	F	CGTCGAGCAGAATTACGACAA
		R	GTGTCGAACGGAGGATCTTTAC
gor	glutathione reductase	F	CGATAACGTGCTGGGTAAGAA
		R	GATCAGGATATGATCGGCAGTG
*trx*A	thioredoxin	F	GACACGGACGTACTTAAGGC
		R	GTCAGCGATCTCATCCAGAATC
*trx*B	thioredoxin reductase	F	GTGATACCCAGAACACCGATAA
		R	CCGGACTGCACTTTGATGTA
rpoB	DNA-directed RNA polymerase subunit beta	F	CCGGAAGGCACTGTTAAAGA
		R	CACGCTCAGTACCGTTGATAA

2 *SI* Figures

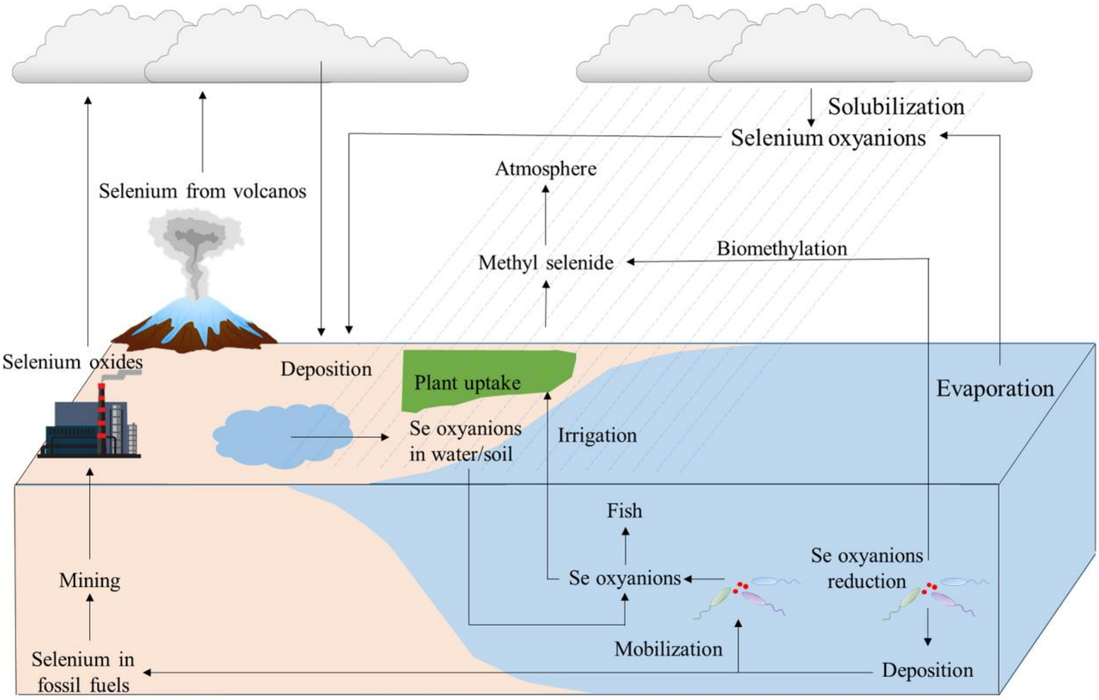

Figure S1. Global Se cycle in nature. Geologic and anthropogenic sources release Se into the environment as selenate. Selenium, an essential element, is assimilated from selenate or selenite by microbes and plants at the base of the food web and subsequently by animals. Selenium is then assimilated into organoselenides in living organism. The decomposition of dead organisms releases Se back into the environment. Mining operations, combustion of fossil fuels, agriculture, and volcanic eruptions release Se into the atmosphere, soil, and water in soluble forms. Microorganisms play a key role in the cycling of Se compounds in nature.

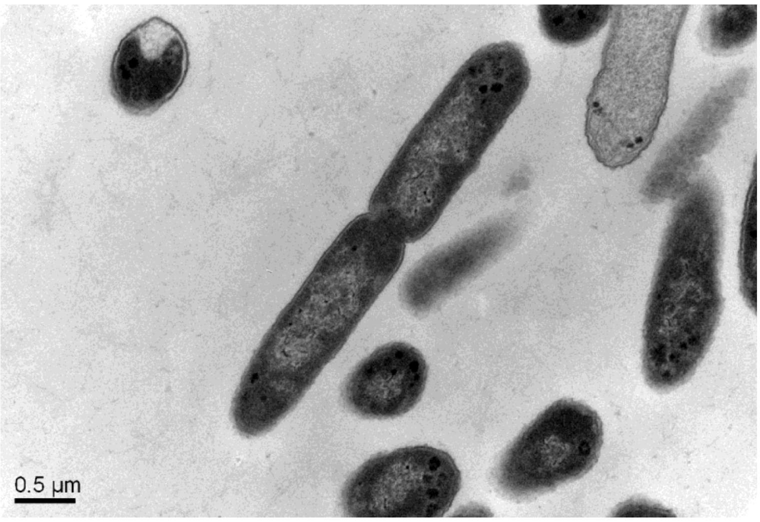

Figure S2. TEM image of *E. cloacae* Z0206 grown without the presence of selenite.

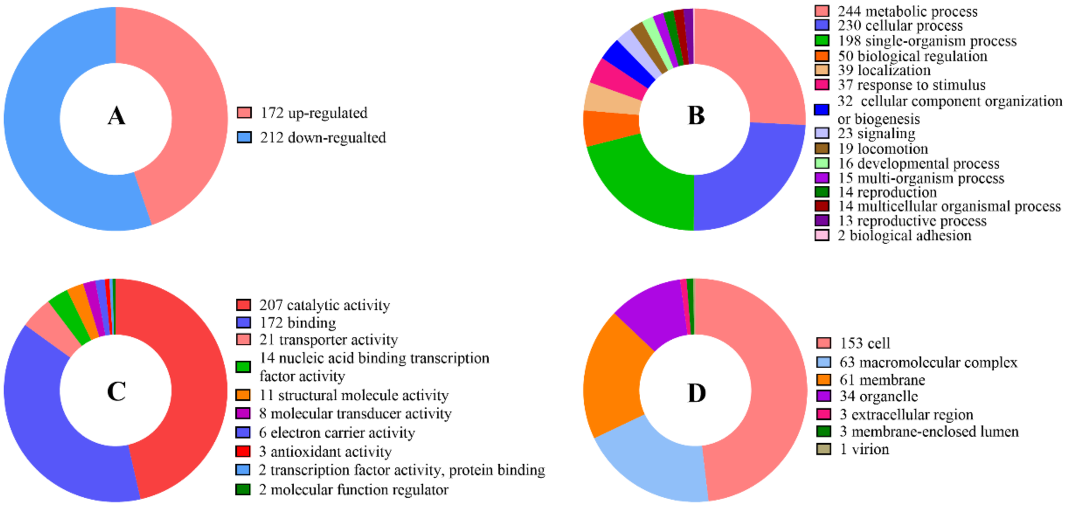

Figure S3. Gene Ontology annotation of differentially expressed proteins identified through iTRAQ analysis in *E. cloacae* Z0206 in response to selenite. (A) Number of proteins differentially regulated as a result of selenite treatment. (B) The biological process distribution of identified proteins. (C) The molecular function distribution of identified proteins. (D) The cellular component distribution of identified proteins.

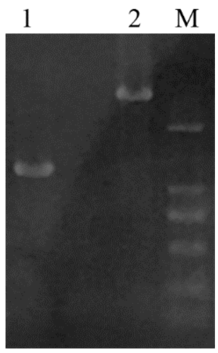

Figure S4. Validation of *frd* gene mutation. The length of *frd* gene PCR product of wild type strain is 2881 bp (lane 2), whereas, the length of that of Δ*frd* is 1183 bp (lane 1). (M, DL2000 DNA Marker)

3 *SI* Tables

3.1 Table S1. Protein quantification of iTRAQ analysis

3.2 Table S2. Effect of selenite on the antioxidant protein expression of *E. cloacae* Z0206

Description	Fold	Significance
Glutathione reductase	1.08	0.38
Glutathione synthetase	1.07	0.41
Glutathione-disulfide reductase	1.06	0.50
Thioredoxin	1.04	0.64
Thioredoxin reductase	1.02	0.84
Thioredoxin-dependent thiol peroxidase	1.01	0.92
